# Herbert Spencer, Sociological Theory, and the Professions

**DOI:** 10.3389/fsoc.2019.00077

**Published:** 2019-12-10

**Authors:** John Offer

**Affiliations:** School of Applied Social and Policy Sciences, Ulster University, Coleraine, United Kingdom

**Keywords:** Herbert Spencer, professions, service users, complexity and agile agents, social organism and spontaneous cooperation, inheritance of acquired characteristics

## Abstract

This article presents new insights into Spencer's theoretical sociology as he applied it to the professions and professional institutions, which he discussed extensively, particularly in his *Principles of Sociology*. The first part of this article notes the main conceptual insights which he established and aligns them within the wider context of a re-reading of Spencer's sociology. Particular attention is paid to the “social organism” and the spontaneous cooperation of social individuals in society (with each possessing “social self-consciousness”). This part also reappraises Spencer's account of the emergence of “professionals” and their distinctive “cunning, skill, and acquaintance with the nature of things,” which professionals have brought to bear on what has been experienced in the ordinary social lives of people as complexity or the unfamiliar in the world. The subsequent discussion focuses on, first, a retrieval of Spencer's theoretical stance on the *activities* of the professions, and on work and conditions in general, and, second, on reviewing some of the major resonances which his work has with practical problems and the associated theoretical issues concerning the sociological understanding of professional/service-user interaction in social life today.

## Introduction

Recent studies on Spencer have produced significant insights about how to interpret his complex ideas afresh, permitting them to be seen in a more substantial and systematically linked conceptual context^1^. In an earlier article the present writer provided a general survey of what new accounts have achieved in terms of additional theoretical coherence to the understanding of Spencer. The present article has a different primary focus, a reassessment of his substantial body of work on the professions and professional institutions. It has been an overlooked strength of Spencer that he nuanced his abstract sociological thinking about aspects of social life with micro-level everyday observation (as argued by Turner, 1985, Ch. 8). One interest which Spencer had in particular was professionals and professional institutions.

In the interests of clarity, however, it is necessary to cover very briefly again a little of what was covered in more detail in the earlier article, chiefly in relation to what Spencer meant by the “social organism” and the place in societies of the spontaneous cooperation of social individuals, where each possessed a sense of “social self-consciousness.” This sense of “social self-consciousness” itself, for Spencer, was brought about by a mechanism of change arising through social and psychological adaptation to circumstances and the inheritance of acquired characteristics. These are matters which will be explained in the first main section.

Spencer's interest in professions must have dated back at least to his years as a young man from 1837 to 1846, when he experienced first-hand the professional responsibilities associated with a brand-new frontier of the art of civil engineering: for, as an early participant in some of the first railway schemes in England as they were constructed or projected, he was in at the birth of a revolution in communications. The primary focus here, then, is a reappraisal of what Spencer says on the professions, professional institutions and work more generally (as it is situated within his theoretical framework), and how these contributions have implications for certain practical problems and theoretical issues in the understanding of facets of professional life today. The discussion deals in particular with such subjects as Spencer and professional self-interest, his views of “justice” and professionals, and his comments on the nature of “work” in a general sense. The discussion also, looking back, shows how his mechanism of individual and social change is one constituent of a cascade of ideas that is itself related to the contemporary social science focus on the complexity of interaction in general and between service users and professionals in particular. This focus highlights “agile” and knowledgeable agents, negotiating pathways through complex landscapes of threats and opportunities, and how they interact with professionals and others implementing policies from social and public policy (interpreted broadly), who are of course agents too, but now ones whose roles may have changed quite recently to a more reactive mode. In short, the brief but relevant and updated account of Spencer as a social theorist dealing with the professions, which forms the first part of the article, prefaces a reinterpretation of his account of changes in the professions, grounded in the action of innovative agents rather than the reified “functions” and “structures” in social life that have often been presumed to be his principle concerns.

## Key Aspects of Retrieving Spencer

In previous publications (2010, 2019), I have discussed the reasons why it is *not* very useful in turn to describe Spencer as a social Darwinist, to regard his general developmental theory of change (which he called a theory of evolution and which including individual and social human life) as having a *central* role in understanding how he interpreted substantive matters such as “professional institutions,” and to accuse him of “atomic individualism”^2^. It can be more rewarding to give prominence to other features. The present discussion is largely concerned with aspects of social life in what he called “industrial” societies, since these were the societies on which Spencer mostly concentrated in writing on professional life. Spencer contrasted “militant” with “industrial” structures. Militant structures are geared for aggression, with strong central and coercive control of the individuals in a society. Industrial structures, however, are geared for peace, so “industrial societies” (societies in which industrial structures predominate), have been described as having a “spontaneously generated a loosely coupled mode of societal organization” (Dingwall and King, 1995, p. 20, now also reprinted in Dingwall, 2016; see too Dingwall, 2004, p. 8–9). In such societies, social controls are “decentered,” that is to say the controls operate at an everyday level, being concerned with interchanges between private individuals in pursuit of private or mutually-agreed ends, not with a direction from the center of such interchanges toward collective purposes. In this way, boundaries between whole societies and their environments, and hierarchies established between their various constituent social elements or groupings can possess a porous quality^3^.

This article begins by unpacking the key statement that Spencer's industrial form of society has “spontaneously generated a loosely coupled mode of societal organization.” Sociologists still connect the idea of the social organism with Spencer. Although he used the expression in 1843 in his Letters collected as *The Proper Sphere of Government*, and in *Social Statics* (Spencer, 1851), it was “The Social Organism” (Spencer, 1860) that clarified what it meant to him. The essay was often later used by Spencer as a reference point to elucidate the meaning of “society”^4^. At that time the enormous variety of forms which biological organisms exhibited in the world, including “compound individuality,” was exciting scientific interest, which Spencer followed. The burning question was how could a society be envisaged in this highly charged context? In a broad but real sense a society *had* to be part of “nature” (it was not after all “supernatural”)^5^.

Neither any particular society nor societies in general was assimilated to any one known individual organism or set of organisms. In every edition of his *Principles of Sociology*, Spencer declared that ‘(t)he social organism, is not comparable to any particular type of individual organism, animal or vegetal (Spencer, 1876, p. 613, in the 3rd edition, Vol. 1, 1893, p. 580). There was a material difference peculiar to the “social organism”: “while in the body of an animal only a special tissue is endowed with feeling, in a society all the members are endowed with feeling” (Spencer, 1860, p. 276). This was a pivotal feature of social life in Spencer's conception of a society as organism, for, if in individual bodies, “the welfare of all other parts is rightly subservient to the welfare of the nervous system, whose pleasurable or painful activities make up the good or ill of life; in bodies-politic the same thing does not hold.” In a political body- a society- where all members have consciousness, corporate life “must therefore serve the lives of the parts” (Spencer, 1860, p. 276–277). The idea of the social organism was “accompanied by transcendent differences” when compared with individual organisms, since a society was an “aggregate of individuals distributed over a wide area” (Spencer, 1871, p. 411). In the process, Spencer grasped that his understanding of the social organism was entirely different to Comte's holistic version (Spencer, 1904, p. ii, 465)^6^.

Spencer recognized the connected point that the members were mobile, or “locomotive” (Peel, 1971, p. 178). How to understand “structure” in societies has always exercised sociologists, but since the neglect of Spencer is widespread it is unsurprising that he was overlooked as a predecessor in Elder-Vass's account of the causal powers of structures in social life. The mechanisms which produce the causal powers on the part of some social structures, says Elder-Vass, do not depend “on spatially specific physical relations between their parts.” These structures are what Elder-Vass describes as “spatially disarticulated” (Elder-Vass, 2010, p. 200). In fact, however, the rudiments of this feature which is germane to “intersectionality” were already well-understood by Spencer; its relevance to social analysis today is a topic to be addressed later.

For Spencer, the “units” (individuals) in society become subject to “actions and reaction between the community and each member of it, such that either affects the other in nature” (Spencer, 1893, p. 11). Individuals adapt to each other and to the society over time, and to external conditions, and the changes made in turn lead to further adaptations, helical in nature. The characteristics acquired are inherited by future generations, according to his theory of evolution, thus forming the heart of the changes involved in “social evolution.” “Character” (or “human nature”) is not fixed but is modified as it adjusts to surrounding conditions. His primary mechanism of organic and social change was pre-Darwinian and consciously derived from Jean-Baptiste Lamarck's idea of the inheritance of acquired characteristics, or functionally produced changes. Darwin had not ruled out entirely this mechanism, but his evidence for what he referred to as “natural selection” to account for species change, what would later be interpreted as the successful survival of genetic variations in an environmental struggle for existence, was in the process of eclipsing it following the publication in 1859 of *On the Origin of Species*. Although Spencer did accept a place for Darwin's natural selection, in 1886, however, he was moved to write in defense of the continued salience his mechanism, especially in individual and social life: “if we admit the inheritance of functionally-produced changes, we are justified in concluding that this inheritance of functionally-produced changes has been not simply a co-operating factor in organic evolution, but has been a co-operating factor without which organic evolution, in its higher forms at any rate, could never have taken place”(Spencer, 1886, p. 424)^7^.

It is not in fact a surprising matter that in our participation in ordinary social life we showed capacities to change, to react and act with knowledge and innovation, not least through the form of professional activity, as will be discussed later. If Spencer had written earlier that a progress in social life was “logically certain,” as he did in *Social Statics* (Spencer, 1851, p. 64), by the *Principles of Sociology* he had forsaken that belief, as will be shown in the next Section. What was and in a measure remains a stumbling block, however, is the idea that the accumulation of these changes should in biological terms be *inherited*^8^.

According to Spencer, a society grows through economic and other acts of spontaneous cooperation by gregarious and social individuals, who are themselves displaying what is called a “*social self-consciousness”* (Spencer, 1859, p. 140–141; Spencer, 1873, p. 291. See too Peel, 1971, p. 217)^9^. In his *Principles of Psychology* Spencer portrays the possession of social self-consciousness as an awareness that the well-being of each person is “bound up with the well-being of all,” resulting in turn in the “growth of feelings which find satisfaction in the well-being of all” (Spencer, 1872, ii, p. 609). In societies in which “industrial” and thus peaceful social relations predominate, the principal job of the community itself through the government is to protect the equal freedom of all citizens in order to be able to adapt to circumstances. As a society coheres through spontaneous cooperation, and as a division of labor emerges, it starts to exhibit a *relatively* stable set of functions and allied structures. Functions and structures are *mutable*, they are malleable according to the changing and varied contents of what the spontaneous cooperations of individuals are in practice attempting to create. Compared with an individual organism, the structures and functions “of the social organism are obviously far less specific, far more modifiable, far more dependent on conditions that are variable and never twice alike” (Spencer, 1873, p. 58)^10^.

By 1860, then, groundwork behind Spencer's conception of the “social organism” had yielded a strong, interlaced set of arguments, not always credited by critics, then and now. A society was an organism, but one with *singular* characteristics. This reading of Spencer on the “social organism” shines light on the cohesiveness of his thought on “the social,” and on the nature of sociology too (there was no “rule” which stated that an organism *must* be seen as a single collectivity). Societies were “organisms,” a constituent of the natural world, but *sui generis* in their units, structures and functions. For Spencer people and societies find “ways of cooperating with each other” and that they initiate jointly by developing “new ideas and practices” (Dingwall and King, 1995, p. 16), an outcome which follows logically from the fundamental and original features of his thought.

## “Professional Institutions” in Spencer's *Principles of Sociology*

Acts of spontaneous cooperation between social individuals can be regarded partly as an adaptive response to what they experienced as complexity or the unfamiliar in the world being encountered. Viewed in this way, Spencer was in the forefront as a sociologist who was confronting the challenge to understand social life as involving people who were making sense of and negotiating ways through complex landscapes, although as it happens he has been largely unrecognized as such^11^. Despite the observations of the previous section, it remains possible that reservations might still be raised on the legitimacy of presenting Spencer in this way, or that the themes in question are to be strongly associated with Spencer. A likely source is the influence of a still commonly-held view that Spencer is definitively associated with a theory of evolution in general and social evolution in particular that is directional and lineal in nature. For if true, Spencer could lack the salience to contemporary sociological concerns being implied. However, Spencer himself clarified his position. Immediately after his most detailed remarks on professions in his *Principles of Sociology* were written, he went on, in the same volume, to disown with conviction that the alleged position on evolution was one he occupied. About evolution in general and social evolution in particular, he stated: “Evolution does not imply a latent tendency to improve, everywhere in operation. There is no uniform tendency from lower to higher, but only an occasional production of a form which, in virtue of greater fitness for more complex conditions, becomes capable of a longer life of a more varied kind” (Spencer, 1896, p. 599).

With that reminder now in place, this article moves on to show that Spencer's focus, on understanding social life as involving social individuals confronting challenging and complex terrains, shapes his work on the actions of professionals and professional institutions in particular. In the event, it is worth noting that this discussion is also providing a rare opportunity to revisit at least a part of the substantive aspects of his sociology. Spencer observed in his general introduction to professions, that the mark of the modern professionals' strength draws on a legacy from the “cunning, skill, and acquaintance with the nature of things” which gave “the primitive priest or medicine-man influence over his fellows.” That kind of entrepreneurial power, says Spencer, “is augmented by those feats and products which exceed the ability of the people to achieve or understand; and he is therefore under a constant stimulus to acquire the superior culture and the mental powers needed for those activities which we class as professional” (Spencer, 1896, p. 184).

Spencer's own main references to professions, as has been noted, occur in his chief sociological book the *Principles of Sociology*, which contained a landmark discussion of “Professional Institutions” (in Volume 3, of 1896). Since the first volume first appeared in 1876 and the second volume in 1882, “Professional Institutions” was late in the sequence of the component Parts, and late in Spencer's own life. Only the “Industrial Institutions” Part was to follow, completing the third and final volume. The previous Parts were the “Data” of sociology (on early man), the “Inductions” of sociology (including “society” as an entity), followed by “Domestic Institutions” and “Ceremonial Institutions,” in turn leading to “Political Institutions” and “Ecclesiastical Institutions,” in both of which Spencer argued were the origins of the professions.

In “Professional Institutions,” Spencer discussed the development and secularization of professions, as well as individual professions, dealing with Physician and Surgeon; Dancer and Musician; Orator and Poet; Actor and Dramatist; Biographer, Historian, and Man of Letters; Scientist and Philosopher; Judge and Lawyer; Teacher; Architect; Sculptor; and Painter.

Professions emerge by being differentiated over time from the general regulation of social life sustained and managed by the “politico-ecclesiastical” agency:

No group of institutions illustrates with greater clearness the process of social evolution; and none shows more undeniably how social evolution conforms to the law of evolution at large. The germs out of which the professional agencies arise, forming at first a part of the regulative agency, differentiate from it at the same time that they differentiate from one another; and, while severally being rendered more multiform by the rise of subdivisions, severally become more coherent within themselves and more definitely marked off (1896, p. 311).

Spencer's conception of the functions of professional institutions accords with his general conception of the relationship between a society and its members: the maintenance of the life of a society, an “insentient organism,” is considered “only as a means to the ultimate end—maintenance of the lives of its members, which are sentient organisms” (Spencer, 1896, p. 179).

Spencer views a society as not a “manufacture” but a growth, and this includes the development of the professions. Social arrangements come about not by changes “contemplated by rulers” (Spencer, 1896, p. 315), not by design, but by changes attributable to “the unprompted workings of this organized humanity” (Spencer, 1896, p. 317):

So unconscious are men of the life of the social organism that though the spontaneous actions of its units, each seeking livelihood, generate streams of food which touch at their doors every hour—though the water for the morning bath, the lights for their rooms, the fires in their grates, the bus or tram which takes them to the City, the business they carry on (made possible by the distributing system they share in), the evening “Special” they glance at, the theater or concert to which they presently go, and the cab home, all result from the unprompted workings of this organized humanity, they remain blind. Though by its vital activities capital is drafted to places where it is most wanted, supplies of commodities balanced in every locality and prices universally adjusted—all without official supervision; yet, being oblivious of the truth that these processes are socially originated without design of anyone, they cannot believe that society will be bettered by natural agencies. And hence when they see an evil to be cured or a good to be achieved, they ask for legal coercion as the only possible means (Spencer, 1896: 316-17).

There is an echo here of the economist Richard Whately, whom Spencer had earlier in time often cited, who had written similarly in his *Introductory Lectures on Political Economy* (Whately, 1832) and had depicted economic exchange as “catallactics.” For Spencer, governments are blind to social life as “catallaxy” (though this is not a word adopted by him)^12^. Through the passage of time, occupations have become so specialized that the labor of each person, satisfying some needs of others, has had their own needs satisfied by the work of hundreds of others. Here, then, is a reiteration of “spontaneous cooperation” as a cornerstone of the “social organism,” and thus of society itself, and the sub-text of professional life.

Given the breadth of activities covered in Professional Institutions, only a few examples of Spencer's comments can be chosen. He commonly illustrates “differentiation” growing up within professions (thus there are, for example, musical performers, professors and teachers of music, and holders of examination passes and of degrees in music; and also differentiation between the many local musical societies and the music colleges, “with their students, professorial staffs, and directors” (Spencer, 1896, p. 214). He illustrates “integration” as achieved though “a periodical literature,” in the case of music, journals “devoted to reports and criticism of concerts, operas, oratorios, and serving to … maintain the interest of the teachers and performers” (Spencer, 1896, p. 214).

In the discussion of Science as a profession Spencer naturally introduces the growth of specializations within subject areas such as biology, but it is noteworthy that in Philosophy he made no mention of “friction” between different “schools” of thought. Spencer occupied a position which embraced utilitarianism and an orientation toward social science, whereas by the 1880s Idealist modes of philosophical thought about how to discover fundamental values and knowledge were in the ascendant in Britain, concerned to question those very developments as heralds of a dehumanizing materialism. These years were witnessing acute differences over fundamental principles between Spencer and Idealists such as T H Green, Bernard Bosanquet, Henry Jones, David George Ritchie (and “New Liberals” in the Liberal Party as well). The key differences were over how best to achieve social reform, the responsibilities of the state and the meaning of concepts such as “the good” and “positive freedom.” The distinctive manner in which Spencer had delineated the social organism and emphasized cooperation and “social self-consciousness,” a self-consciousness in each citizen “of the state of the aggregate of citizens” (Peel, 1971, p. 2017), tended to be side-lined or misrepresented in favor of “society” conceived as a holistic “moral organism.” For Ritchie, for example, Spencer's individual was depicted in a skewed and exaggerated way, “as if he had a meaning and significance apart from his surroundings and apart from his relations to the community of which he is a member” (Ritchie, 1885, p. 646, also in Offer, 2000, Vol. 4, p. 106).

In Professional Institutions, Spencer eschewed making any remark that this trend toward Idealist philosophy was tantamount to harking back to the orthodoxies associated with militant and coercive social relations^13^. Perhaps he judged that a concise developmental account of professions in general was an inappropriate place to display personal philosophical divergences, even if they were profound.

Spencer viewed professional specialization as adaptive for individuals and societies. It encouraged them to prosper and allowed a more competitive edge because, as Dingwall and King remarked, “of the way in which it introduced more flexibility, more cooperation, and more space for innovation” (Dingwall and King, 1995, p. 16). Spencer had emphasized more the creative role of the professions “than do most modern analysts” (Dingwall and King, 1995, p. 19). For Everett Hughes, Spencer regarded the “elaboration” of the roles of professions as “the essential feature of civilized society” (Hughes, 1960, p. 54). Such accounts correctly conclude that Spencer understood professionals in industrial societies as market actors, acknowledging that they enhance the quality of life by having services to offer which are innovative and substantive. This viewpoint, Dingwall and King observe, “is an important corrective to some of the nihilism of the sociology of the professions in the 1960s and 1970s, when it often seemed that professions were merely…auxiliary organs of capitalist power” (Dingwall and King, 1995, p. 19).

Relationships between the professions and the state were of constant interest to Spencer. This dimension also appealed to Dingwall and King who concluded that a virtue of Spencer's focus was to open the way “to a more convincing analysis of the relations between the state and the professions” (Dingwall and King, 1995, p. 21), as compared to Abbott (1988), who concentrated on relationships *between* professionals (these themes are developed in the later sixth Section “Spencer, professionals and their users.” Dingwall and King stretch credibility, though, when describing Spencer as delivering a “paean for *laissez-faire*” (Dingwall and King, 1995, p. 22), thus disregarding his rebuttals of the expression as applied to him, made in a rejoinder to T H Huxley in Spencer's “Specialized administration” of 1871 (Spencer, 1871, p. 438), and, quoted here, in 1893 in “Evolutionary Ethics” (Spencer, 1897, p. 115).

I am not aware that anyone has more emphatically asserted that society in its corporate capacity must exercise a rigorous control over its individual members, to the extent needful for preventing trespasses one upon another. No one has more frequently or strongly denounced governments for the laxity with which they fulfill this duty. So far from being, as some have alleged, an advocacy of the claims of the strong against the weak, it is much more an insistence that the weak shall be guarded against the strong, so that they may suffer no greater evils than their relative weakness itself involves. And no one has more vehemently condemned that “miserable laissez-faire which calmly looks on while men ruin themselves in trying to enforce by law their equitable claims (*Ethics*, § 271).”

Dealing with the protection of the liberty of all in social relationships providing that none should infringe the like liberty of another is the proper sphere of government. To work to ensure “justice” in this sense is for Spencer an expectation placed upon the conduct of professionals and professional institutions (Mingardi, 2015). This liberal conception of “justice,” which Spencer consistently held as a writer on social matters, limited the role of government in peaceful, “industrial” states, to the protection of the equal liberty principle. Spencer should have written more systematically about forms of power in “industrial” social life but he did recognize them, and his awareness led him to a specific inference about how face-to-face interactions between a range of professions and citizens should be conducted. In addition to the state's responsibility to protect a citizen's person “and punish criminal aggression on him,” there is the responsibility “to administer civil justice to the citizen free of cost” (Spencer, 1891, p. 660–61). Given that legislators raise taxes from us, Spencer asks rhetorically whether the government takes up the cause of the poor man to defend him “against the aggressions of his rich neighbor.” In its failure to defray this cost of maintaining civil order it commits the sin of omission. Government abandons the complainant “to the tender mercies of solicitors, attorneys, barristers, and a whole legion of law officers” (Spencer, 1843, in Offer, 1994, pp. 3–57, p. 51). In his later *Principles of Ethics* Spencer renewed the complaint. The State may guard citizens “against offenders classed as criminals,” but if a citizen “is defrauded of an estate” it “turns deaf ears to his complaint, and leaves him either to bear the loss, or run the risk of further and perhaps greater loss in carrying on a suit and possibly appeals” (Spencer, 1910, vol. 2, p. 210). Had the penalties been clear and certain at the outset, the majority of civil offenses “would never have been committed” (Spencer, 1910, vol. 2, p. 211).

Spencer had identified a serious hindrance in the way of permitting ready access to sound and practical professional advice and guidance for ordinary citizens at the point of need. He understood that his concept of “justice,” a pivotal component of the conditions required for the conduct of social life, made it imperative that the chances should be maximized for citizens to have available, promptly and freely, the services of the “family” of professional skills in the area of the law, thus enabling cases of injustice to be replaced by justice as a matter of urgency.

## Professions and Self-Interests

“Professional Institutions” does not exhaust what Spencer said on professions. Whether in his early *The Proper Sphere of Government* (Spencer, 1843) or the later *The Man versus The State* (Spencer, 1884, in Offer, 1994, pp. 61–175), a recurrent target was the “interested motives” by “certain medical men” to advocate “enactments for the preservation of the public health,” irrespective of need (Spencer, 1843, p. 56)^14^. In *Social Statics*, the critique embraced a comparison between medics and the clergy: the fear that unauthorized preachers could spread false doctrines “has its analog in the fear that unauthorized practitioners may give deleterious medicines or advice…(t)here is an evident inclination on the part of the medical profession to get itself organized after the fashion of the clericy” (Spencer, 1851, p. 375). Nervousness about Messianic tendencies reappeared in Professional Institutions: “the priesthood of medicine persecutes heretics and those who are without diplomas. There has long been, and still continues, denunciations of unlicensed practitioners, as also of the “counter-practice” carried on by apothecaries …. a constant tendency to a more definite marking off of the integrated professional body” (Spencer, 1896, p. 199). Perhaps with the best of intentions, myopic professionals desired compulsory legislation, depriving individuals of their liberty and encouraging dependence on the state, at the price of slowing the process of social evolution by which individuals (and by inheritance their offspring) can progressively adapt to their circumstances. Against this compulsory enlightenment the antidote was what Spencer called “a systematic study of natural causation as displayed among human beings socially aggregated” (Spencer, 1884, p. 123. See too Spencer, 1873, p. 66–7, 81–2).

Spencer had interpreted ecclesiastical institutions as one of the original sources of later, largely secular professionals. An earlier section of the *Sociology* had discussed the prospects for churches and priests. He interpreted religious beliefs and practices as in general long-term decline, with the future of “Ecclesiastical Institutions” involving churches and priests transitioning from the calling of dogmatic theology to offering counsel and advice in more nuanced tones. In an evolving world standards of morality were a relative matter: “The ideas of right and wrong, now regarded as applying only to actions of certain kinds, will be regarded as having applications coextensive with actions of every kind. All matters concerning individual and social welfare will come to be dealt with, and a chief function of one who stands in place of a minister, will be not so much that of emphasizing precepts already accepted, as that of developing men's judgments and sentiments in relation to those more difficult questions of conduct arising from the ever-increasing complexity of social life” (Spencer, 1896, p. 157–58)^15^. In this case, of course, self-interest might indicate joining a profession such as social work. Spencer seems to have not considered any case of professions or of professional institutions in decline beyond those associated with religious observation (for a recent study of the decline of the profession of actuary see Collins et al., 2009).

## Spencer and the Wider World of Work

Aspects of work and employment which impinge on professional activities, in particular the trades unions, working conditions in general and unpaid work, attracted Spencer's attention. His critical comments derive from his equal freedom principle (justice). Trades unions can resort to coercion to demand obedience from their members and obstruct piece-work practices (Spencer, 1910, ii, p. 279–281; Offer, 2010), and in their name strikers had resorted to violence and hence injustice against employers (Spencer, 1910, ii, p. 294–96). However, justice is also a weapon on the side of unions. “Judging from their harsh and cruel conduct in the past,” Spencer writes, “it is tolerable certain that employers are now prevented from doing unfair things which they would else do” (Spencer, 1896, p. 542). Clearly pertaining to wages and health at work, these are remarks seldom credited to Spencer: Wiltshire, for example, accused him of “inveterate hostility” to trade unions (Wiltshire, 1978, p. 141; 161).

On some general working conditions Spencer's condemnation is unequivocal (as Peel noted, 1971, p. 216). The advances in machinery in factories “has proved extremely detrimental” in mental and physical respects for the health of the operatives (Spencer, 1896, p. 515). The wage-earning mill worker may exemplify free labor, but “this liberty amounts in practice to little more than the ability to exchange one slavery for another” …. “The coercion of circumstances often bears more hardly on him than the coercion of a master does on one in bondage” (Spencer, 1896, p. 516. Spencer's misgivings about wage labor and injustice, and his support for trade unionism, receive further discussion in Weinstein, 1998, p. 201–207).

Spencer was well-aware that, in ordinary social interaction, there was much activity showing the “fellow feeling” which was integral to social life, as opposed to the perspective of “atomic individualism” of which, as has been noted earlier, some critics have charged him:

Always each may continue to further the welfare of others by warding off from them evils they cannot see, and by aiding their actions in ways unknown to them; or, conversely putting it, each may have, as it were, supplementary eyes and ears in other persons, which perceive for him things he cannot perceive himself: so perfecting his life in numerous details, by making its adjustments to environing actions complete (Spencer, 1910, p. i, 254).

Moreover, he captured for his readers in some sympathetic detail the intricate dynamics of “private beneficence,” or informal care, arising when families or neighbors undertake to tend or nurse ill or frail family members or acquaintances. Private beneficence had moral qualities that made it preferable to state beneficence, but he was disquieted by the disproportionate burdens of care which were performed by women and the restricted opportunities which followed in its wake (on Spencer on this aspect see Offer, 1999). Spencer's comments on unpaid work pivot around “beneficence” in the *Principles of Ethics*. Beneficence is altruism over and above the demands of “justice.” Spencer's concern with “beneficence” as against “justice” and its demands here signified that he was continuing a tradition familiar from *The Theory of Moral Sentiments* by Adam Smith^16^.

Voluntariness in the shape of voluntary organizations conformed to Spencer's ideas about social development. He welcomed the organized but spontaneous voluntary action (the “provident beneficence”) which was forthcoming when ordinary persons, who had acquired surgical and medical knowledge, stepped in to provide help to sufferers before the arrival of professional help (Spencer, 1910, ii, p. 361). Hiskes (1983) traces the treatment of Spencer's idea of social individuals and his liberal idea of community since his day, criticizing some libertarian sources, including Robert Nozich's *Anarchy, State, and Utopia* (1974), for ignoring the altruistic attitudes and motivations of people in social life which Spencer had described in detail. In addition, Spencer's familiarity with *The History of Cooperation in England* of 1875 by G J Holyoake and *The Cooperative Movement in Great Britain* of 1891 by Beatrice Potter made him amiably disposed toward organized cooperative ventures in production: we were witnessing the “germ of a spreading organization” (1896, p. 564)^17^.

Any discussion of Spencer and the wider world of work has to consider at least briefly Spencer's reference to a “third” type of society beyond his distinction between “militant” and industrial' types of societies. In the first and subsequent editions of the first volume of the *Principle of Sociology* (Spencer, 1876) he predicted that when the industrial form was more fully developed, societies will use the results not exclusively for material aggrandizement, but for carrying on “higher activities” as well. The new type is indicated by “inversion of the belief that life is for work into the belief that work is for life” (Spencer, 1893, p. 563). The changes to be expected are “the multiplication of institutions and appliances for intellectual and aesthetic culture” (Spencer, 1893, p. 563). In 1882, the third type became the theme of his speech delivered in New York, warning his audience of the barrenness of an obsession with work (Spencer, 1904; Shapin, 2007; Werth, 2009, ii, p. 387–409). Again, it is significant that Spencer frowned on *laissez-faire* without boundaries.

## Spencer, Professionals, and Their Users

Spencer identified professions by their role or function in social life in his own evolutionary-based narrative (his distinctive, non-Darwinian, theory of evolution). The professions had an innovative power in social life: once “the defense of life, the regulation of life and the sustentation of life” have been achieved, then the professions in general foster the “augmentation of life” (Spencer, 1896, p. 181). It is interesting that in a new research Tracey Adams, studying professional self-regulation in Canada, points explicitly to Spencer's importance as an analysist of professions, underlining his emphasis on the special insight or “expertise” of professionals, particularly in their “institutional roles” (Adams, 2018, p. 29). As was noted earlier, Spencer had singled out professions for their honed “acquaintance with the nature of things” (Spencer, 1896, p. 184).

Some important points arise around his emphasis on the idea of adaptation to circumstances by individuals and societies. As has been discussed already, the key mechanism of change in the organic world in general for Spencer is the inheritance of acquired characteristics following adaptation to circumstances, and by social individuals in particular (and thus on to the communities which they form). Dingwall and King noted that Spencer “is not thinking simply of the material or biological environment, but of selection pressure from other individuals or societies” (Dingwall and King, 1995, p. 16). Thus the scene is set in which the *potential dynamism*, but also precariousness, of the nature of the professional/service-user interface becomes a central topic.

As indicated earlier, Dingwall and King discussed Abbott's application of an ecological perspective to the understanding of “the processes by which boundaries are drawn and redrawn between the elements in an ecological system,” and to the sequencing of the development of professions. By contrast, they argue, Spencer has as a main interest interchanges “both between the system elements and across the boundary that distinguishes the system from its environment” (Dingwall and King, 1995, p. 21): the flaw in Abbott's methodological approach as against Spencer's was that it seemed to exclude the growth of the various layers of state/professions relationships.

Since 1995 and the Dingwall and King publication, Abbott's work drawing on ecology has been enlarged upon in approaches invoking co-evolution and complex adaptation, as described and explained in, for example, Room's *Complexity, Institutions and Public Policy: Agile Decision-Making in a Turbulent World* (Room, 2011). Other recent work covering the quintessential Spencerian theme of professionals, change, and complexity has been explored fruitfully by the French sociologist Florent Champy (although in this case as far as I am aware without making a specific connection to Spencer). In commenting on studies of professionals, he notes “professionals are sometimes confronted with a level of complexity that brings some irreducible uncertainty in their work. It is impossible to know with scientific certainty what should be done and what exactly would result from any action once completed. Working with people (e.g., in medical practice, social work, teaching) is emblematic of this type of difficulty” (2018, p. 1). However, Champy goes on to suggest that “theorizing complexity and its effects on professional work has never been a priority for the sociology of professions.” He adds that sociology has paid “little attention” to how professionals “manage to accomplish their work, the concrete difficulties they encounter or their dissatisfaction concerning the outcome of their work” (2018, p. 1).

In this context Room (2016), while referring to a later publication than was available to Dingwall and King by Abbott (2001, ch. 5), hints at a less constrained interpretation of Abbott's work than they held. Given the concerns of the present article, Room's ideas in particular need further discussion. To be clear, it is no longer the case that professions are in a bounded system, simply in a struggle between each other (if ever that were the case) for the openings in which their members can earn their crusts, but that there is “open access” to information for all, which risks redundancy for all; a new “survival of the fittest,” to use Spencer's own coinage^18^. In “uncertain and foggy landscapes” (or environments), the relationships with the state which pertain for professionals need to as much figure in “adaptive walks” as with their relationships to other active agents, including individuals, families and communities. All these social actors are likely to be seeking positional advantage (Room, 2016, p. 100–101). The basic premise is that we live in a world of increasingly agile and informed choice-making agents [“queens” in Le Grand's (2006) terminology] who are potential users or non-users of a range of services from policy-makers and professionals. Policy-makers and professionals, with any traditional assumptions of a monopoly of professional wisdom placed in jeopardy, have to “compete” with these agents on what must be conceptualized as a complex, uncertain and turbulent landscape if their advice is still to be perceived as sound^19^. Room included a discussion of a population group at risk of social exclusion and poverty in this scenario, applying findings from research on lone mothers by Millar and Ridge (Ridge, 2007; Millar and Ridge, 2009). Households which are at risk of losing some welfare support through benefit reforms are, Room says, “re-weaving the *bricolage* of their resources and relationships, in an effort to resist exclusionary pressures” (Room, 2011, p. 258). The householder, as “agile institutional entrepreneur,” actively reworks “the complex web of formal and informal social affiliations,” including any given employment openings, in which they are enmeshed (Room, 2011, p. 257). The parts played by family, friends and neighbors come into the mix, and if a job is available the question becomes whether or not taking the job is likely to dovetail adequately with the children's childcare needs.

At the same time, social institutions and the professionals associated with them can be expected to adapt to the terrain with which they are newly confronted. The risks associated with their decisions may create turbulence, with unequal costs falling on the least agile households. Powerful groups, sometimes including professionals, may intervene, “to ensure as far as possible that their interests are protected, their position consolidated and the costs of uncertainty displaced onto others,” and thus “occupying the future” (Room, 2016, p. 194). Implicit gender assumptions about individual members of families and how their incomes and expenditures in fact contribute to family as opposed to individual life may need to be confronted, especially in times of crisis (Walby, 2015—the previous Section made a relevant link to Spencer is this regard). There is in fact a considerable and highly relevant research literature on informal care and the interface between family members who are informal carers on the one hand and professionals and policy-makers who devise their community care support on the other^20^.

The discussion here suggests that a logical continuum appears to have been neglected which in a significant manner connects the operation of Spencer's mechanism of change in social life, the adaptation to circumstances by social individuals through spontaneous cooperation, with the recent work on “agile” agency, adaptive walks, the negotiating of complex landscapes and reaching “successful” outcomes^21^. In the interests of both the history of social theory and the reflexive development of the recent theorizing in relation to professionals, policy-makers and service-users, Spencer's mechanism and his own applications of it to interpreting professional and social life, and apparent resemblances between them and the more recent investigations, suggest an affinity which merits more sustained enquiry and acknowledgment in the future.

## Summary and Conclusion

This article began by reviewing some recent reinterpretations of the sociological work of Spencer which have substantially helped to retrieve his reputation by clarifying its central ideas, emphasizing his own interpretation of the social organism and idea of the social self-consciousness of individuals. The narrative explained the overall structure and theoretical cohesion of Spencer's sociological thought, and then how he came to apply it to professionals and work in general. Spencer stressed that through their specialization, privileged “acquaintance with the nature of things” and skill, and “cunning,” professionals introduced more flexibility, more cooperation, and more space for innovation into social life.

It then covered Spencer's more detailed understanding of “professional institutions” in his *Principles of Sociology*, complemented by material from other sources. Although helpful insights were noted from Dingwall and King (1995) their view that Spencer was an uncritical supporter of *laissez-faire* was challenged as against the evidence. His distinctive concern with relationships between the state and professions, a liberal interpretation of equal freedom or “justice” and his argument for the administration of civil justice to the citizen to be free of cost were discussed; and also the theme of self-interest and professional life, itself a lifelong preoccupation for Spencer. The established church might be losing power, but there was an aspiring establishment order, the “priesthood” of medical men, about to win additional powers from governments and a new bureaucracy to implement them^22^. Aspects of the wider world of employment and work in Spencer's sociology were raised, including the injustices represented by unreasonable working conditions, just criticisms made by trade unions, the significance of the role of women in unpaid domestic care and the costs to them, and the prospect for professional life in a future, post-industrial form of society.

The discussion of Spencer and relationships between professionals and users suggested how accounts of Spencer's ecological approach to the evolution of the professions can be updated. A key part of Spencer's mechanism of change in social life was the adaptation to circumstances by social individuals, and that mechanism resonates really quite deeply with some important recent work relating to professionals, policy-makers and service-users, which focuses on the theoretical and practical significance of skills associated with ideas of “agile” agency and negotiating complex “landscapes,” and reaching “successful” outcomes^23^.

To end it will be helpful to highlight just two of the substantial range of themes of conceptual and theoretical significance in particular that occupied Spencer which have special resonance in making sense of professional activity and everyday social life today. The first theme is that of the centrality of the adaptability to circumstances in Spencer. He becomes a potential catalyst to lead us into fresh questions about the nature of the relevant changes in the “social self-consciousness” of citizens about the state of other citizens (perhaps involving changes in the balance of power), in times when shifting events and nuances are altering experiences as agents-as-users (with their expectations) and of professionals/policy-makers (with their expectations) when they are interacting reflexively in complex situations. The other theme, often overlooked in discussions of Spencer, is the need for freedom to access readily the *administration* of justice^24^. The redress of injustice is itself a potential source of wellbeing which seems sometimes to be underrated within social and public policy studies. It also matters because this freedom is still an overlooked but central theme of the normative side of *Spencer's* analysis of social life, in general outside the scope of the present study.

Spencer had displayed an intuitive grasp of the first theme in his writings on evolution in general and on sociology and psychology in particular a century and quarter ago, and the second was a radical coda. Taken together and with the other points discussed they were the key concerns arising from his work on professionals and professional institutions. Spencer on professions and service users deserves a better fate than oblivion on these kinds of matters. But as Spencer himself observed, “fashion is an accompaniment of the industrial type (of society) as distinguished from the militant” (Spencer, 1891, p. 209).

## Data Availability Statement

All datasets generated for this study are included in the article/supplementary material.

## Author Contributions

The author confirms being the sole contributor of this work and has approved it for publication.

### Conflict of Interest

The author declares that the research was conducted in the absence of any commercial or financial relationships that could be construed as a potential conflict of interest.

## Footnotes

^1^Part of the complexity of studying Spencer as a sociologist is that important insights into understanding his sociology are gained not only from *Social Statics*, the *Study of Sociology* and the *Principles of Sociology* (and many of his essays), but also from wider framework in which his ideas were set out: *First Principles*, the *Principles of Psychology*; the *Principles of Biology*; and the *Principles of Ethics*. On occasion there were changes of substance between first edition and later editions.

^2^McCann (2004, p. 125–26) is also a key source arguing against seeing Spencer as a individual atomist.

^3^For Spencer, late Victorian Britain strictly counted as semi-militant, semi-industrial (Offer, 2019).

^4^It has important links to his essay of 1857 (Spencer, 1857), “The ultimate laws of physiology” (re-published in his *Essays* as “Transcendental physiology”).

^5^Some parts of the discussion in this section are dealt with more extensively in (Offer, 2015).

^6^The exact nature of Spencer's understanding is also underlined by (Taylor, 2007). On this specific matter I think Durkheim understood Spencer's position. However, on some other matters Durkheim misunderstood Spencer's own thought. Durkheim was incorrect when he described Spencer's sociology as displaying (as Durkheim himself wrote), a “narrow utilitarianism and utilitarian egoism,” and “moral poverty,” giving as a reason for his statement that “it is only too clear that all social life would be impossible if there did not exist interests superior to the interests of individuals” (in Lukes, 1969, p. 20). Spencer's important idea of individual “social self-consciousness” is introduced later in this Section of my article. By contrast, Gabriel Tarde strongly criticized Durkheim's view there is a constraint that is “normal” associated with “reflection which causes man to understand how much richer or more complex and permanent the social being is than the individual being” (Durkheim, 2013, p. 98), but misunderstood the precise and novel delineation of what Spencer meant by the “social organism” (see Tarde, 1898, p. 78–79, “Sociology,” in Clarke, 1969).

^7^The complex and nuanced way, mostly overlooked, in which Spencer sought to accommodate Darwin's evolutionary mechanism of “natural selection” (to his own satisfaction, and with a “modification”) into his much more general, all-embracing theory of evolution in the *Principles of Biology* is documented in Offer (2014). The inheritance of acquired characteristics is described as the mechanism of “direct equilibration,” and what *Spencer* calls “natural selection” is described as “indirect equilibration.”

^8^Note that Meloni, a social theorist working on the historical, conceptual, and political implications of aspects of the life sciences, has remarked that “in the surprisingly brief period since 2000 we have witnessed an impressive rehabilitation of ideas that only three decades ago we would find bizarre or unacceptable: basically, that there might be more than DNA transmission in biological heredity” (Meloni, 2018, p. 138). See too Gissis (2005), on the running together or inoculation of biological heredity and cultural inheritance in Spencer's *Principles of Psychology*.

^9^In Durkheim's criticisms of Spencer's understanding on “society” and “individual” I have found no evidence that he was aware of the significance of Spencer's concept of “social self-consciousness.” In Spencer's world, once there is a self-consciousness of others the space for collective life is created. In terms of Bird's analysis of liberal individualism, Spencer's introduction of the idea of social self-consciousness should help to free him from accusations of “atomic” individualism (Bird, 1999, p. 206, 208). This was the misplaced nub of Durkheim's critique of Spencer on the foundations of “society” (Offer, 2019).

^10^Yet note too that Spencer denied free-will in the *Principles of Psychology*, in all editions of the book (Offer, 2006, p. 60).

^11^In his *The Study of Sociology*, written to persuade skeptics of the need for the subject, Spencer referred to the “extremely-complex phenomena which Sociology deals with” which demands a complex intellectual faculty having the “plasticity that can receive with ease” the “countless combinations of social phenomena” (1873, p. 145). Using the example of then the new automatic Walter Press, employed for printing the *Times*, Spencer paints a picture of the myriad and non-linear social events and processes which were drawn together to make its invention possible. The phenomena which made it possible were complex, “but there is mostly an absolute unconsciousness that there are any such complex phenomena to be grasped” (1873, p. 132).

^12^There is no firm evidence that Freidrich Hayek was influenced by Spencer but there is some likeness (Smith, 1981, 2013, p. 203; McCann, 2004). In his “Sociology and neoliberalism: a missing history,” however, Gane makes no mention of how Hayek has similarities with Spencer on spontaneous cooperation, and on altruism (Gane, 2014, p. 1101).

^13^As he was to record in his correspondence in 1902. See (Duncan, 1908), p. 457–8.

^14^See also Spencer's essay “The morals of trade,” in which there are examples of the misdemeanors of solicitors and barristers (Spencer, 1859, p. 139).

^15^On Spencer's contrast between “absolute” and “relative” ethics see Taylor (2007), p. 116–123.

^16^Smith's own concern was noted by the botanist, lawyer and writer, Bicheno (1824, p. 150), who distinguished “beneficence” from “benevolence”: benevolence “may signify either the *principle*, or that intuitive instinct or impulse which excites in us sympathy toward the distressed, and a desire to relieve and communicate happiness; or, it may signify the *exercise* of the principle in the actual communication of happiness. This, in order to distinguish it from the former, has been frequently called *beneficence*” (1824, p. 78). Bicheno was important as a champion of voluntary beneficence and critical of statutory poor law provision, particularly in Ireland (McGauran and Offer, 2017).

^17^Spencer and Holyoake were contemporaries and on friendly terms. Spencer was close to the Potter family, encouraged Beatrice's interest in social science, and figured in her autobiographical *My Apprenticeship*, (Webb, 1926) and *Our Partnership*, (Webb, 1948). She married Sidney Webb in 1892, but despite her Fabian socialism and particular enthusiasm for the work of health professionals she and Spencer remained on good terms. Since it also cuts across some preconceptions of Spencer, note that he personally intervened to try to secure Williams and Norgate as publisher (in the event successfully) for Booth's *Life and Labor of the People*. In a letter to Williams (Jan. 4 1889) wrote: “This letter will be handed to you by my friend Mr Charles Booth, whose name you have probably seen in the newspapers in connection with statistics of labor in the East-end. He has I know bestowed much trouble in giving a true account of a very involved matter, and his results are recognized and quoted as authoritative. He wishes to give a permanent form to these results, and proposes to negotiate with you as publisher. I shall be glad to hear that you have come to a satisfactory arrangement” (Letter to Spencer to Williams, Knox College *Illinois, Bookfellow Foundation Collection)*.

^18^Spencer first used the expression, as a tailored alternative to Darwin's “natural selection” in the *Origin of Species* of 1859, in his first volume of his *Principles of Biology* of 1864. However, to be clear here, Room stresses that “it is not sufficient to treat such selection mechanisms as “blind forms of “self-organization”. Powerful actors struggle purposefully to organize and reshape these processes and the direction of technological and institutional change, so as to reinforce and defend their own positional advantage. Here there is an endless dance” (Room, 2016, p. 104).

^19^Faced with such complexity Champy has advocated the merits of the “technique” of exercising “practical wisdom” by professionals, rather than the mechanical use of protocols and rules (Champy, 2012, 2018).

^20^The roots of this body of research go back to the 1970s (Offer, 2006). *The Client Speaks* by Mayer and Timms (1970) is a classic text. When in the 1970s Robinson wrote in *In Worlds Apart: Professionals and Their Clients in Welfare State* that a major factor in “troubles” in “professionals-client relationships is the encapsulation of each in sharply differing subjective worlds” (Robinson, 1978, p. 2) he was reporting the general findings of the times. As mentioned earlier the topic (as “beneficence”) was discussed by Spencer. An example of recent relevant research in Europe is (Broese van Groenou and De Boer, 2016).

^21^Room points out that it is not sufficient to say “that a particular invention is “complex”, merely in having a number of interrelated components in a “complicated jumble”: what matters are the non-linear dynamics to which such connections can lead” (2016, p. 143). The first paragraph of the section in this discussion, “Professional Institutions” in Spencer's “*Principles of Sociology*,” including Note 12, shows how Spencer seems to meet this criterion.

^22^His late essay “Sanitation in theory and practice” accused “the sanitary class,” emerging “since Chadwick's day,” of exaggerating “the evils to be dealt with while tacitly exalting its own members” (Spencer, 1901, p. 156).

^23^It is important to note a further dimension here, pertinent to the passing on of acquired characteristics, particularly of potential relevance to professions in the area of health and social care. Meloni and Müller have concluded that “together with a growing number of social scientists, humanities scholars and life scientists, we propose that epigenetic research and, particularly, work on such a sensitive topic as transgenerational epigenetic inheritance needs interdisciplinary exchange and collaboration to thrive responsibly” (Meloni and Müller, 2018, p. 8).

^24^The process of redressing injustice through the state often remains, at least in the United Kingdom, one which incurs substantial costs and delay for the appellant in tribunals. In tribunals legal aid “is available on a more limited basis than it is for the ordinary courts” (Mullen, 2010, p. 399).
